# Altitudinal variation in leaf morphology and functional traits of sea-buckthorn (*Hippophae rhamnoides*) in Gilgit region, Pakistan

**DOI:** 10.1038/s41598-025-25019-y

**Published:** 2026-01-05

**Authors:** Sadia Hakeem, Zulfiqar Ali, Muhammad Abu Bakar Saddique, Muhammad Ali Sher, Sabah Merrium, Martin Wiehle

**Affiliations:** 1 Institute of Plant Breeding and Biotechnology, MNS University of Agriculture, Multan, Pakistan; 2 Ayub Agricultural Research Institute, Faisalabad, Cotton Research Institute, Multan, Pakistan; 3 Department of Plant Breeding and Genetics, University of Agriculture, Faisalabad, Pakistan; 4 Programs Department, Islamic Organization for Food Security, Mangilik Yel Ave. 55/21 AIFC, Unit 4, C4.2, Astana, Republic of Kazakhstan; 5 Section of Organic Plant Production and Agroecosystems Research in the Tropics and Subtropics, University of Kassel, Steinstrasse 19, Witzenhausen, D-37213 Germany; 6 Centre for International Rural Development, University of Kassel, Steinstrasse 19, Witzenhausen, D-37213 Germany

**Keywords:** Climate change, Leaf functional traits, Leaf wettability, Peltate trichomes, Leaf architecture, Water use efficiency, Ecology, Biodiversity

## Abstract

Sea-buckthorn is a multi-purpose plant that provides food, feed, fuel, and medicine. Climate conditions affect its adaptability more than the terrain characteristics. Limited research has focused on its leaf functional traits important for climate adaptation. This study characterized seventy sea-buckthorn accessions from five locations in Gilgit region of northern Pakistan (2444–3172 m.a.s.l) for leaf-functional traits (leaf angle, hairs, rolling, groove, and wettability – summarized as leaf traits and physiological traits including stomatal conductance (gs), leaf relative water content (RWC), transpiration (E), water use efficiency (WUE) and photosynthesis (A)). Leaf surface structures were observed under a scanning electron microscope. Majority of accessions at Misgar (higher altitude) showing 0–20% inward leaf rolling, medium groove, and semi-erect leaf angle had higher E, gs, and lower WUE, RWC, moderate A, and hydrophilic leaf surface. Comparatively, most of accessions at middle altitudes (Passu) indicated adaptive leaf characters i.e., semi-droopy leaves, light leaf groove, 20–40% leaf rolling, moderate trichome density with hydrophobicity (> 90°) and high drop rolling efficiency (< 15°), and had higher A (8.9 ± 1.4 µmol CO_2_ m^−2^ s^−1^), WUE (4.8 ± 0.4 mmol CO_2_ mol^−1^ H_2_O), and fruiting (~ 67%). Moreover, the positive association of WUE with fruit yield indicated high photosynthetic productivity of such accessions. Umbrella-shaped peltate leaf trichomes were 151–175 μm long, with higher densities on abaxial surface, appressed to the epidermis and two layers with overlapping rays-shields. Stomatal density was higher on the abaxial surface, mostly covered by the trichomes. This study provides theoretical backgrounds of an ideotype suited for climate resilience supporting sea buckthorn germplasm conservation.

**Supplementary Information:**

The online version contains supplementary material available at 10.1038/s41598-025-25019-y.

## Introduction

In high-mountainous ecosystems, parameters like solar radiation, temperature, humidity, and nutrient availability, along with other abiotic factors, vary significantly, even within minimal altitudinal differences^1^. The effects of climate change may intensify these variations in vegetation dynamics, distribution patterns, and community structure. Long-term exposure to these climatic extremes exerts a selection pressure on plant species, imparting structural and functional adaptations for survival. For instance, plants show variable adaptability characteristics that are influenced by their genetic makeup, plasticity and agronomic practices to thrive in these environments^2^. Typically, high-altitude plants possess a wide range of morphological characteristics: their leaves are small but highly lignified, developing xeromorphic structures^3^ or the epidermis is thickened and sclerification around vascular bundles occurs^4^. Some also exhibit dense hair-like structures on the leaf and stem, and their stomata are often grooved and protected by small cavities^5^. Although most of these features are genetically fixed, their responses vary with changing environments, particularly with altitude^4^.

Sea-buckthorn (genus *Hippophae* L., family Eleagnaceae) is a nutrient-rich valuable wild plant widely distributed across mountainous regions of Asia including China, India, Mongolia, Pakistan, Türkiye as well as seashores of middle and northern Europe^6^. Owing to its superfruit potential, it can be a valuable source to combat malnutrition by augmenting it in the food basket^7^. Among the 50 to 100 Elaeagnaceae species – its systematics is still under debate—^8^ *Hippophae rhamnoides* (sea-buckthorn) is the most widely distributed across Asia and Europe. It is currently being domesticated and commercially cultivated in Asia, Canada, and Europe^9^. While the value of its products has been commercialized and upscaled in countries like China, India and Mongolia, in Pakistan it is a neglected species. Although it contributes a significant source of income for a few entrepreneurial farmers in the Gilgit-Baltistan regions of Pakistan^10^. Around 86% of the farmers in the region have been reported to own sea buckthorn plants on their land^11^. It is adaptable to poor soils and can withstand a wide temperature amplitude between − 40 to + 40 °C^12^. The phytochemical properties of the leaf^13^ and the fruit^14^ are well characterized. Habitat suitability under climate change scenarios has also been reported^15^, but limited data were found for leaf traits, especially functional traits like leaf attitude, photosynthesis, transpiration, stomatal conductance, density and size, water content, drop rolling efficiency, and epidermal surface properties^16,17^.

Leaf traits, including morphological, physiological, and stoichiometry characteristics, influence the growth, reproduction and distribution of plant species^18^. Leaves regulate ecological processes like gas exchange, photosynthesis, transpiration, and nutrient cycling^19^. Leaf visual and morphological traits like colour, shape, angle/orientation, and degree of marginal dissection or leaf groove, are not only species indicators^20^, but are also subjected to climate differences^21^. The variation in the three-dimensional leaf architecture depends on water availability, the cultivar, and the environment^22^. Features like water-absorbing leaf surfaces, tip-drip, and surface patterning^23^ are known adaptational traits in wet climates^24^, while waxy coatings and leaf curling are utilized for hot temperature mitigation in drier environments^25^. The leaf wettability, represented as the amount of water in the form of rain, dew, fog, and snow retained on the leaf surface, is also affected by the cuticle surface, leaf architecture, and morphology^23^. Leaf wettability has both positive and negative effects on leaf and the plant^26^: while it inhibits gas exchange and photosynthesis^26^, leaches nutrients^27^, and increases pathogen infection^28^, especially at longer periods, it can also increase water availability by direct uptake of water from the leaf surface and suppression of transpirational water losses and may thus maintain the plant’s vigour, improve growth, and increase its survival^23^. For instance, leaf features like leaf hydrophilicity, inclination and rolling^29^ as well as hairs and grooves^30^ are suitable candidate adaptational traits in wheat under semi-arid conditions and can thus be employed as traits in other species of commercially still low importance.

Physiological traits like photosynthesis, stomatal conductance, and transpiration are in turn good indicators of water and CO_2_ exchange and water use efficiency^31^. Thus, knowing morpho-physiological traits can be a significant contribution to understanding the adaptational mechanisms of a plant to future climate conditions. Additionally, unravelling leaf functional traits at a regional level is important to understand the functional background and environment-plant interaction at the global scale and to develop prediction models^31^.

The current study characterized sea-buckthorn germplasm based on leaf functional and physiological traits that can be considered as candidate traits for the adaptation and climate resilience of sea-buckthorn. The study tested the hypotheses that a combination of leaf traits: (i) enhances instantaneous photosynthetic water use efficiency (WUE_inst_) by optimizing physiological parameters including stomatal conductance (gs), net photosynthesis (A), and transpiration rate (E); and (ii) helps adaptation across altitudinal gradients through anatomical features like trichomes, leaf wettability, and drop rolling efficiency. These features can help to select the promising accessions suited to environments with limited water availability, such as the water-scarce low-land regions of Central and Southeast Asia.

## Materials and methods

### Sampling area and plant material

**Fig. 1:**
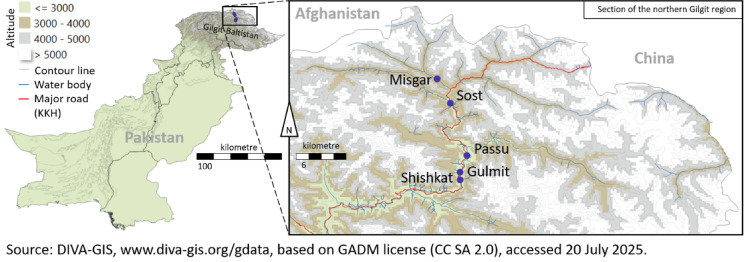
Geographical coordinates of the five sea buckthorn populations studied in Gilgit region, Pakistan.

Five locations of different altitudes from Shishkat to Misgar of the Gilgit region in the northern Karakoram range of Pakistan were chosen (Fig. 1, Table S1). The region’s terrain ranged from valley bottoms to steep hillside. Soils from all locations had a gravelly and loamy texture with generally lower nutrient availability at high altitudes. Fourteen accessions were sampled from each location keeping a 100 m distance among accessions to avoid clonal sampling^32^. From each accession, three samples were collected. At least 20 leaves/fruits per sample were collected for technical repeats. The location and altitude were determined using a handheld GPS device (Garmin OREGON-750, accuracy up to 2.4 m, GARMIN^®^ Ltd., New Taipei, Taiwan). According to the Köppen climate classification system^33^, Gilgit region is classified as BSk, indicating an arid (B), steppe (S), and cold (k) climate. During the sampling duration (October 2021), maximum temperature ranged from 14 °C to 27 °C, while minimum temperature ranged from 1 °C to 13 °C (Figure S1). The average relative humidity at 00:00 h and 12:00 h was 69 and 44%, respectively, maximum during mid-October (82–93%). No rainfall was recorded during this month.

### Phenotyping for morphological traits

**Fig. 2:**
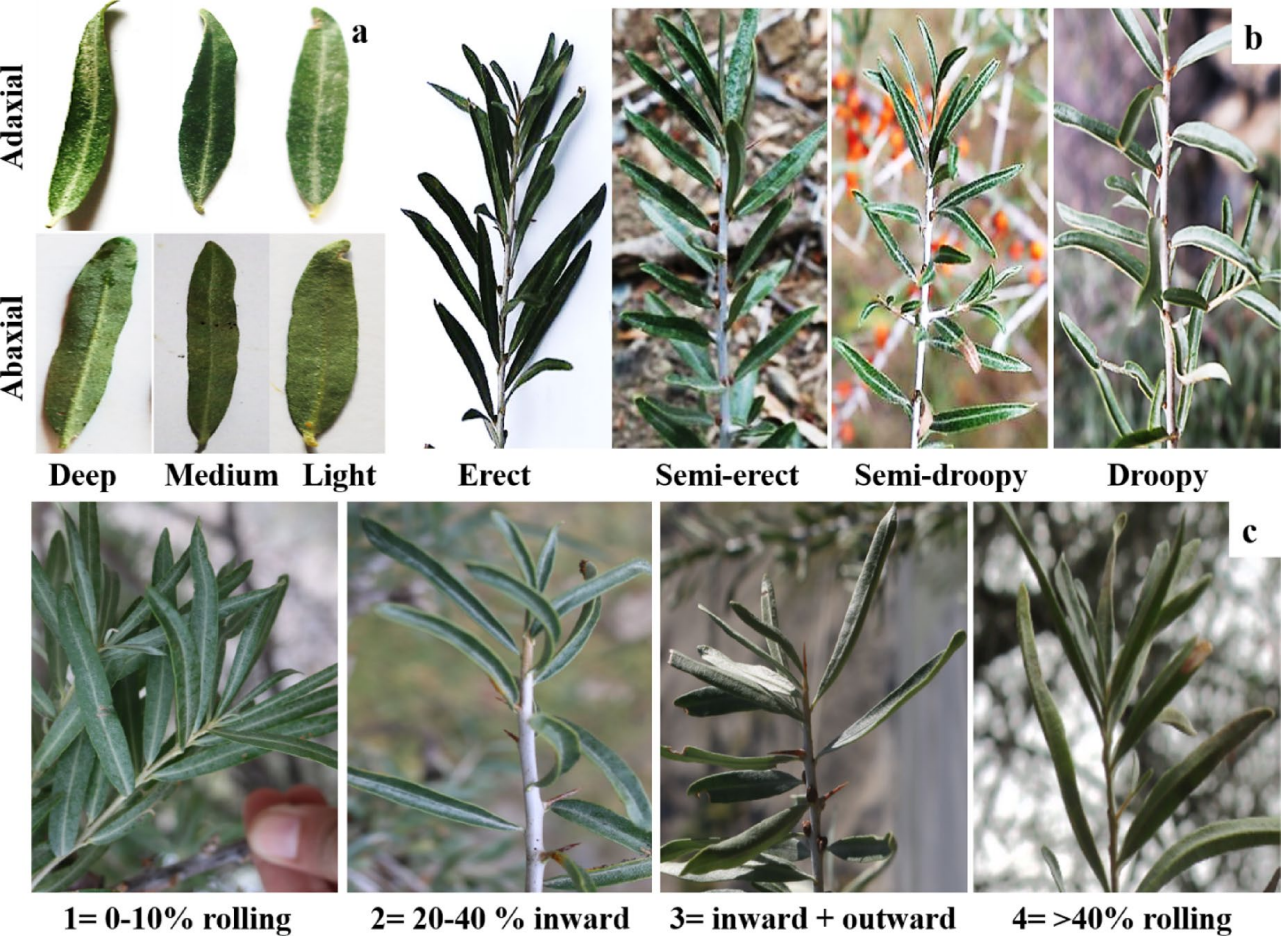
Exemplary photographs of varying leaf traits of sea-buckthorn individuals (*n* = 70) at five different locations in Gilgit region, Pakistan. (**a**) Groove types on abaxial and adaxial leaf surfaces. (**b**) Leaf-to-stem angles. (**c**) Types of leaf rolling behaviour.

**Fig. 3:**
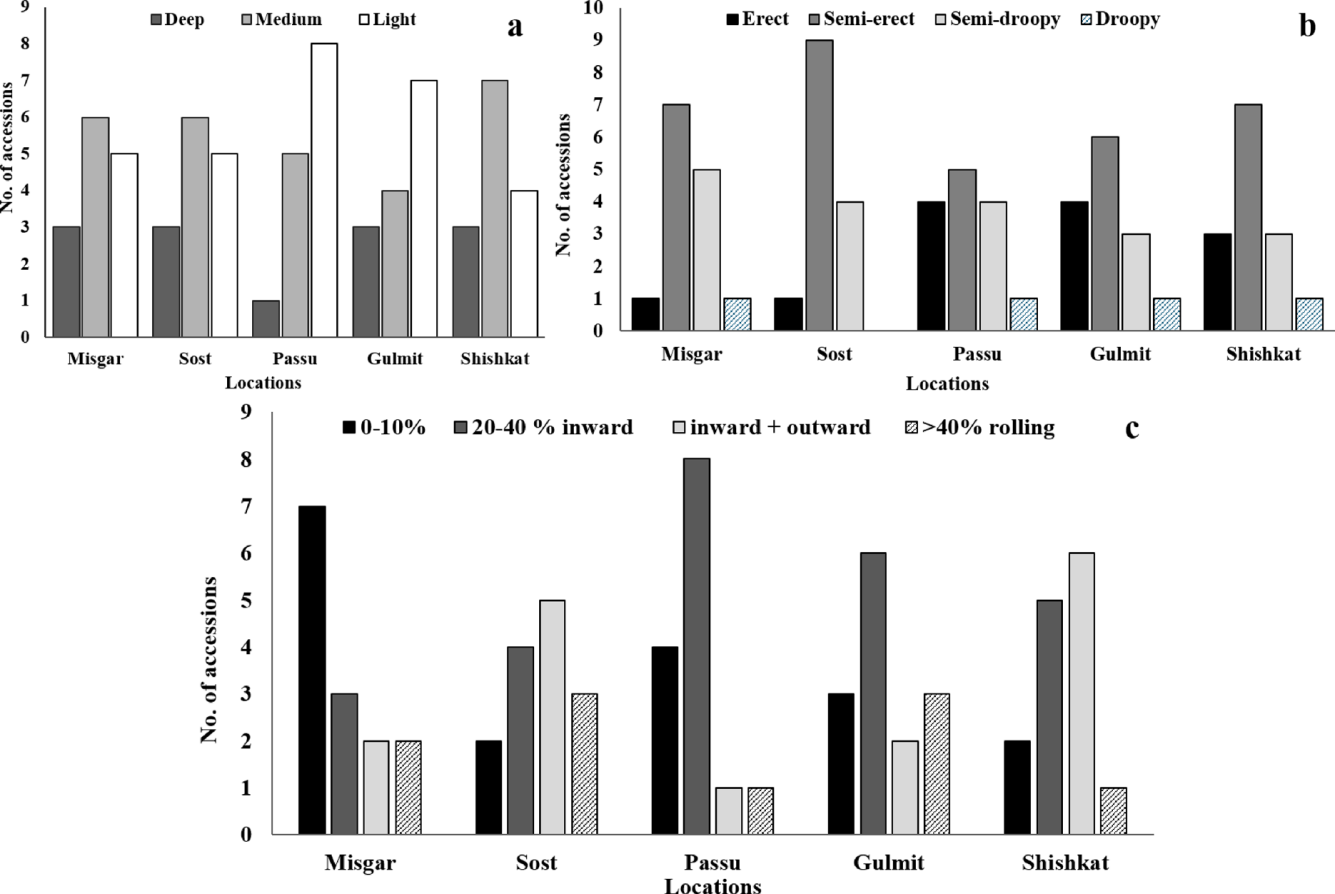
Frequency distribution for the leaf traits in 70 accessions of sea-buckthorn at five different locations. (**a**) groove type, (**b**) leaf-stem angle, (**c**) leaf rolling.

To understand the stature of the leaf, the leaf groove (GT) was categorized into three classes (Fig. 2a) following Hakeem, et al.^30^ while the leaf-to-stem angle (LA) was categorised into four phenotypic classes (Fig. 2b) using the scale by Merrium, et al.^22^. Leaf rolling-being a common indicator of stress and influencing leaf architecture- was quantified based on the percentage of leaf rolling following the scale from Pask, et al.^34^ as well as the direction of leaf rolling (inward, spiral or outside, Fig. 3c). For yield comparison of individual shrubs, fruiting (FS) was measured as a percentage of fruits per individual at the time of data recording^35^, using visual count method of Carevic, et al.^36^ by three different observers.

To indicate the wettability of leaves, static contact angle (SCA) of the abaxial and adaxial leaf surfaces was assessed using a contact angle device (OCA 25, data-physics instruments GmbH, Germany) at room temperature (14–16 °C, 65–73% relative humidity). Per accession, three leaf samples were used, followed by five measurements each on abaxial and adaxial surfaces, resulting in a total of 30 data points. The samples were cleaned from any impurity and dust before performing the measurements. Deionized water was used for the procedure. The SCA was measured by depositing a droplet of 1 µl volume at the dosing rate of 0.05 µl s^−1^ on the abaxial and adaxial leaf surface, respectively. The drop rolling efficiency was assessed by measuring contact angle hysteresis (CAH) based on sessile-drop goniometry method^37^. The CAH was calculated by the difference between the Advancing Contact Angle (ACA) and Receding Contact Angle (RCA). For this purpose, the volume of the drop was increased from 1 to 3 µl s^−1^ for ACA and decreased from 3 to 1 µl/s^−1^ for RCA at a dosing rate of 0.05 µl s^−1^ on both abaxial and adaxial leaf surfaces.

### Measurement of leaf physiological parameters

Physiological parameters including stomatal conductance (gs, mmol H_2_O m^−2^ s^−1^), net photosynthesis (A, µmol CO_2_ m^−2^ s^−1^), rate of transpiration (E, mmol H_2_O m^−2^ s^−1^), photosynthetic water use efficiency (WUE, mmol CO_2_ mol^−1^ H_2_O), were measured using the 80018-3 CIRAS-3 portable photosynthesis system (PP Systems, Amesbury, USA). The data were recorded from the leaf adjacent to the fruit.

The leaf relative water content was calculated using the formula RWC (%) = [(FW-DW)/(TW-DW)] × 100, where FW, DW, and TW represent fresh, dry, and turgid weights of the leaf tissues, respectively^38^. Ten fully-grown leaf samples per accession were weighed for FW, TW (saturated in distilled water, usually for 24 h until full turgidity), and DW (dried in an oven at 65 °C for 72 h until constant weight), and were averaged.

### Scanning electron microscopy analysis for leaf surface structure

Leaf surface, i.e. grooves, trichomes, and stomata, was studied using a Raster Scanning Electron Microscopy (S-4000, Hitachi Global, Tokyo, Japan) at the Institute of Chemistry, University of Kassel, Germany. The dried leaf cross-sections from abaxial and adaxial portions were mounted on stubs and sputtered with platinum (12 nm, Model SCD 005/CEA 035, BAL-TEC GmbH, Witten, Germany). The specimen was observed under vacuum, with an accelerating voltage of 10 kV, at a working distance of 15 mm. The SEM images were analysed using ImageJ software (version 1.52). The length of the rays was measured from the midpoint of the central dome.

### Statistical analysis

All the statistics were performed with R software v. 4.1.2 (R Core Team, 2020) in R Studio. The analysis of variance was performed using the ‘agricolae’ package^39^. For the evaluation and selection of accession for all the traits under study, a principal component analysis was performed with the packages ‘ggplot2’, ‘plyr’, ‘scales’, and ‘grid’^40^. The analysis was conducted by utilizing the scaling and centring features of the package. The relationship among traits was developed using Pearson correlation (package ‘corrplot’)^41^. Box plots were produced using the equisser function (packages ‘tidyverse’, ‘agricolae’, ‘devtools’, ‘esquisse’, and ‘hrbrthemes’). Bar graphs were Excel-generated.

## Results

### Variations of leaf morphological traits across five locations

Overall, 13 (18.6%) of accessions had deep, 28 (40%) had medium, and 29 (41.4%) exhibited lighter leaf grooves (Figure S2). Gulmit and Passu had the highest number of accessions (15; ≥ 50% from each location) with light grooves (Fig. 3a). Thirty-four accessions (49%) showed semi-erect leaf angle (Fig. 3b), while only four accessions (6%) had droopy leaf angle (Figure S2b). Passu and Gulmit showed a high diversity for leaf angle (Fig. 3b). Twenty-six (37%) accessions had an inward leaf rolling, largely found at medium altitudes (Passu and Gulmit; Figure S2c), while eighteen accessions (26%) had no to slight leaf rolling, largely found in Misgar (Fig. 3c). In the most southern village Shishkat, six out of 14 (43%) accessions showed inward-outward leaf rolling (Fig. 3c).

**Fig. 4:**
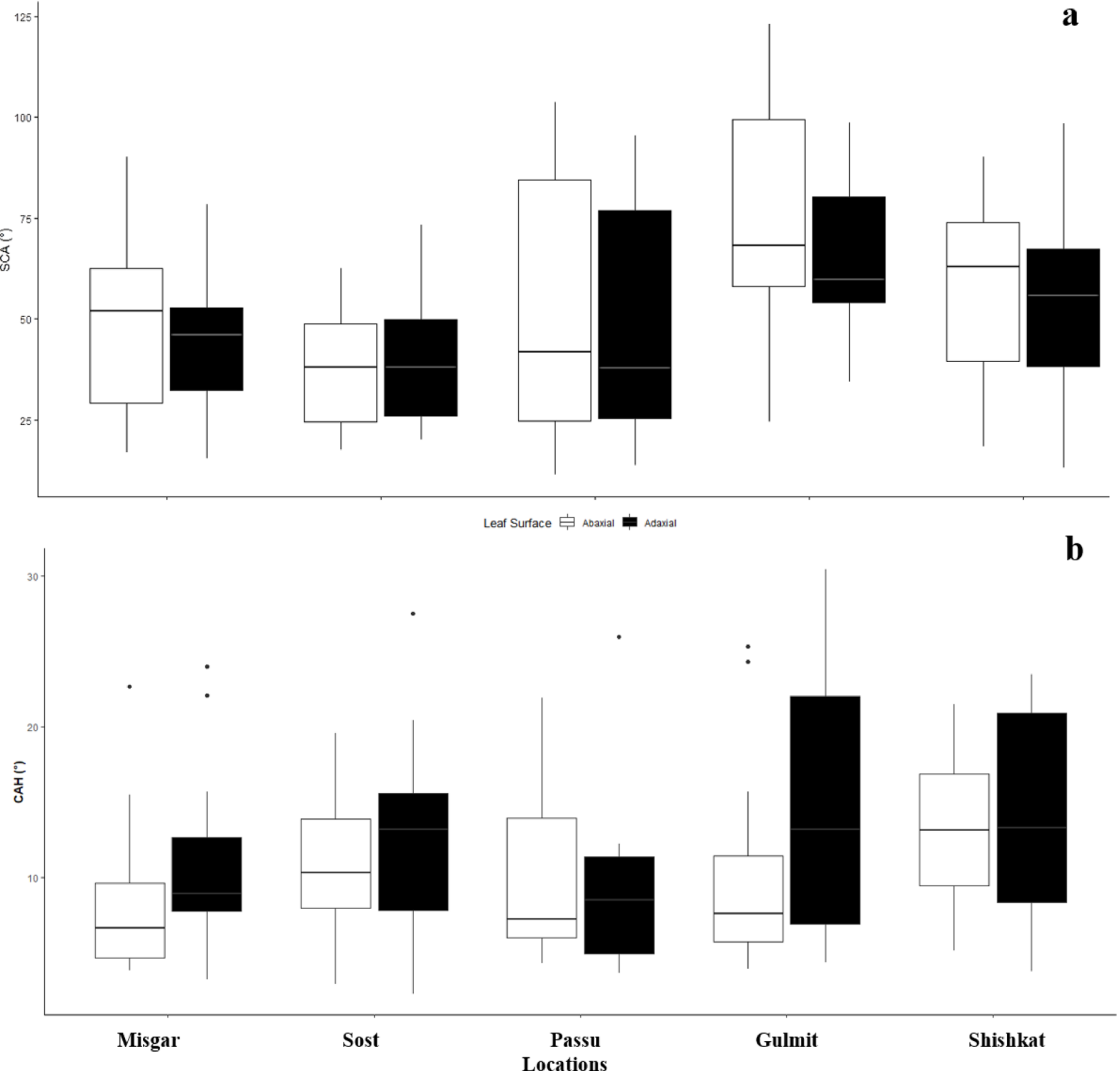
Contact angle on the abaxial and adaxial leaf surface of 70 accessions of sea-buckthorn in Gilgit region, Pakistan. (**a**) Static contact angle (SCA), (**b**) Contact angle hysteresis (CAH); The black dots represent the outlying accessions.

### Leaf surface wettability

Sixty accessions (86%) were classified as hydrophilic (SCA < 90°) to super-hydrophilic (SCA < 40°) while only ten (14%) accessions had contact angle greater than 90° (hydrophobic) either on abaxial or adaxial surface, or both (Fig. 4a). Hydrophobicity trend was higher on the middle altitudes like Gulmit and Passu with 32% accessions (nine out of 28) having hydrophobic SCA (> 90°) on either of the leaf surfaces. Fifty-nine accessions (84%) had CAH less than 15° indicating high drop rolling efficiency, while only 11 accessions (16%) had CAH higher on either abaxial or adaxial surface (Fig. 4b) mostly found in Gulmit and Shishkat.

**Fig. 5:**
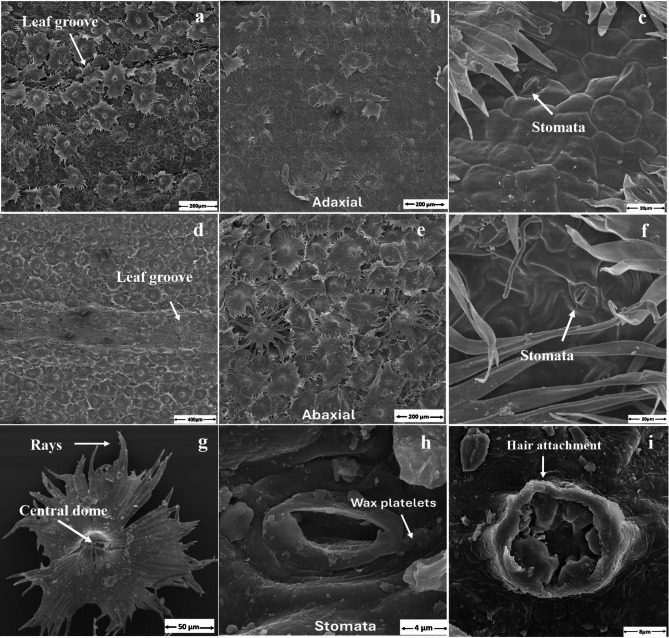
Leaf surface attributes of sea buckthorn observed under scanning electron microscope. (**a**–**c**) Adaxial leaf surface of sea-buckthorn; (**a**) leaf groove, (**b**) Peltate trichomes on adaxial surface, (**c**) magnified view of adaxial leaf surface. (**d**–**f**) Abaxial leaf surface; (**d**) leaf groove, (**e**) Peltate trichomes on abaxial surface, (**f**) magnified view of abaxial leaf surface. (**g**) Single peltate trichome (151 μm diameter) with central dome and rays, (**h**) Stomatal diameter of leaf (4.23 μm), (**i**) Point of attachment of trichome to the leaf.

### Surface structure of sea-buckthorn leaf

The trichomes on sea-buckthorn leaves (Fig. 5a,d,g) ranged from 151 to 175 μm in length. The trichome density was lower on the adaxial surface (21–42 mm^−2^, Fig. 5b) compared to the abaxial surface (61–91 mm^−2^, Fig. 5e) giving lower leaf surface a silvery appearance (Figure S3). Their umbrella-shaped form (peltate) is appressed to the epidermis, has a central dome (28–54 μm diameter), and ~ 50 rays between 94 and 250 μm length and ~ 10 μm width (Fig. 5g). At least two layers of peltate trichomes with overlapping rays-shields were found on the abaxial surface with varying developmental stages (Fig. 5e). The leaf groove was also covered with trichomes, especially the rays of trichome intermingled in the leaf groove (Fig. 5a,d). Leaf epidermal surface was polygon-shaped (Fig. 5c,f). Stomata were found on both surfaces (Fig. 5c,f) with ~ 5 μm diameter (Fig. 5h), but the density was higher on the abaxial surface, mostly covered by the trichomes. Trichomes are attached by a short stalk to leaf epidermis (Fig. 5i). Wax complexes were generally not found in high density and were only rarely observed in a few accessions in the form of platelets.

**Fig. 6:**
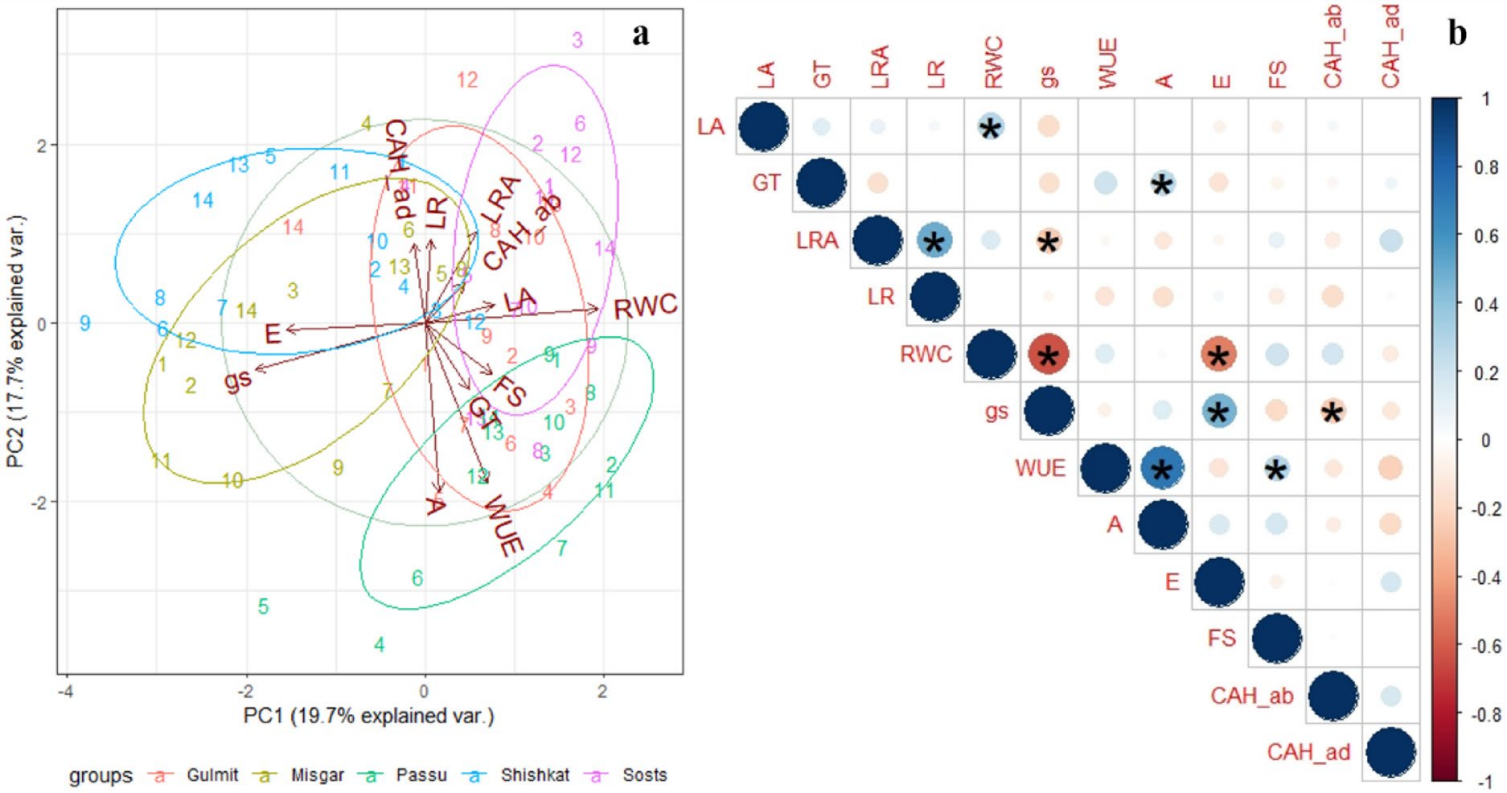
Principal component analysis (**a**) and Pearson correlation plots (**b**) for the morpho-physiological traits among 70 sea-buckthorn accessions across five locations in Gilgit region, Pakistan. Morpho-physiological traits are *LA* leaf angle, *LR* leaf rolling, *LRA* leaf rolling attitude, *RWC* relative water content of leaf, *FS* fruiting (%), *GT* groove type, *A* photosynthesis, *gs* stomatal conductance, *WUE* water use efficiency, *E* transpiration rate, *CAH* contact angle hysteresis, *ab* abaxial leaf surface, *ad* adaxial leaf surface.

### Performance of sea-buckthorn accessions across different regions

The principal component analysis for leaf morpho-physiological traits indicated varying responses dependent on the environment (Fig. 6a). While Gulmit shared most of the leaf traits and fruiting characters with all other populations, the remaining populations showed only minor overlaps. For instance, most distinguishing variables for Passu were A and WUE, while E and gs were the main determinants for Shishkat and Misgar. High values for LRA (leaf rolling attitude) and RWC were separating Sost. Overall, LA, LR, FS, and GT showed lower variations indicated by the shorter vector size. Pearson correlation coefficients (Fig. 6b) indicated significantly positive correlations for most variables, except for RWC which was negatively correlated with gs and E. Similarly, gs showed negative association with LRA and CAH of the abaxial surface.

**Table 1:** Mean values with standard deviation for physiological traits across sampling locations.

Physiological traits	Misgar	Sost	Passu	Gulmit	Shishkat
gs	83.0 ± 21.0	73.3 ± 18.9	80.0 ± 17.0	72.3 ± 10.5	77.5 ± 12.8
A	8.0 ± 0.8	7.2 ± 1.4	8.9 ± 1.4	7.5 ± 1.6	6.4 ± 0.7
E	2.3 ± 0.5	1.8 ± 0.6	1.9 ± 0.4	1.8 ± 0.9	1.7 ± 0.4
WUE	3.5 ± 0.5	3.4 ± 0.6	4.8 ± 0.4	4.2 ± 0.9	3.5 ± 0.4

*gs* stomatal conductance (mmol H_2_O m^−2^ s^−1^), *A* net photosynthesis (µmol CO_2_ m^−2^ s^−1^), *E* rate of transpiration (mmol H_2_O m^−2^ s^−1^), *WUE* photosynthetic water use efficiency (mmol CO_2_ mol^−1^ H_2_O).

## Discussion

Changing climate conditions in sea-buckthorn growing regions have been reported to influence both physiological and morphological traits of the species^42^. The current study indicated significant variations among accessions in terms of leaf structure, surface properties, and physiological properties (Table S2). Accessions collected along an altitudinal gradient showed distinct responses in leaf structural and physiological traits. However, the results support the adaptive leaf behaviour of accessions from medium altitude regions (Passu). These accessions seemed to have better water conservation properties, like moderately rolled leaves (20–40%; Fig. 3c) to maintain WUE and RWC (72–91%), increased leaf angle (semi-erect)^43^ facilitating higher photosynthesis A (7.6–11.6 µmol CO_2_ m^−2^ s^−1^) and WUE (4.2–5.1 mmol CO_2_ mol^−1^ H_2_O; Fig. 6a; Table 1). Mooney, et al.^44^ also observed higher ratio of plants with horizontal/high angle leaves at medium altitudes compared to higher altitudes that had nearly vertical leaf angles. Gemlik olive plants also showed adaptive leaf rolling under restricted water conditions^45^, while in Russian olive (*Elaeagnus angustifolia*) inward leaf curling was observed as a photoprotective response during sunny days^46^.

In general, valley regions of Gilgit (Passu and Gulmit) show increasing trends of average temperatures (1.8 °C per 39 years), while precipitation decreased at < 2800 m.a.s.l by 0.15 mm decade^−1^^47^. This might also be the reason for the moderately to extremely rolled leaves of these accessions. However, the influence of other contributing factors like plant developmental stage, time of the year, and distance from the water body cannot be neglected. These leaf traits (moderately rolled leaves) higher leaf angle properties contributed to higher WUE (4.4 ± 0.8 mmol CO_2_ mol^−1^ H_2_O), A (8.2 ± 1.6 µmol CO_2_ m^−2^ s^−1^), moderate E (1.8 ± 1.6 mmol H_2_O m^−2^ s^−1^), and gs (76.1 ± 14.2 mmol H_2_O m^−2^ s^−1^) on an average for Passu and Gulmit (Fig. 4a; Table 1), in line with the findings of Tangu^45^ who concluded that moderate LR led by anatomical changes maintains WUE in olive. These accessions showed higher fruiting percentages (Fig. 6a), probably due to optimised A and WUE.

In Misgar (highest altitude), 50% of accessions showed 0–10% LR (Fig. 3b) and had higher gs (60–122 mmol H_2_O m^−2^ s^−1^), A (6.7–9.0.7.0 µmol CO_2_ m^−2^ s^−1^), and E (1.6–2.9 mmol H_2_O m^−2^ s^−1^), but lower WUE (2.7–4.0.7.0 mmol CO_2_ mol^−1^ H_2_O; Fig. 6a; Table 1). This leaf behaviour could be expected due to higher rate of precipitation at higher altitudes. The higher altitudes (> 2800 m.a.s.l) receive 800 mm of precipitation in the form of snow and rainfall compared to 200 mm of rainfall at lower altitudes as observed by Hussain, et al.^47^. In water-availability conditions and increased stomatal conductance, a 1000 m.a.s.l increase in elevation doubles the rate of transpiration^48^. Lawson and Blatt^49^ also found that plants with high stomatal conductance have higher photosynthesis rate, but they exhibit lower WUE. Moreover, the semi-erect to erect LA exhibited by these accessions (Fig. 3b) also promotes higher E and gs, as also supported by Casey^50^, who found that leaves are vertically orientated with increased rate of transpiration to cool-off the canopy as a stress avoidance response to solar radiation load in *Quercus velutina* (dyer’s oak). The lower leaf angles (erect) at high altitude regions like Misgar and Sost is also supported by Lovelock and Clough^51^ who found it to be an avoidance mechanism to adapt higher solar radiation levels. Yang, et al.^52^ also reviewed that a negative correlation was found between A and LA meaning that the erect leaf angles with respect to the horizontal plane reduced radiation absorption and thus reduced photosynthetic capacity.

Leaf wettability also supports the adaptability phenomenon as the accessions of the medium to low elevations (Passu and Shishkat) tend to have hydrophobic leaf surface, while overall the sea-buckthorn leaves mostly from Misgar and Sost (higher altitudes) were hydrophilic (Fig. 4a). The leaf wettability properties in the Oleaster family are mostly contributed by the predominance of trichomes. The circular and stalked trichomes known as peltate trichomes, are typical features of Elaeagnaceae (Oleaster) family^53^ and enable (1) a protection against high irradiation^54^ and drought under arid conditions^53^, (2) a fast capturing of water especially for individuals with high trichome density on both abaxial or adaxial leaf surfaces^55^. Bei, et al.^46^ found that the super-hydrophobic nature of the leaf surface in *Elaeagnus angustifolia* (Russian olive, Elaeagnaceae family) promotes air-moisture harvesting. The conical spines on the edges of umbrella-shaped ratchet leaf trichomes collect water, while grooves on the leaf surface help in droplet movement aided by higher contact angles (> 110°) to roll-off the water droplets that are ultimately absorbed by the stomata found abundantly on abaxial leaf surface. Similar trend could also be anticipated for the accessions at middle altitudes (Passu and Gulmit) with hydrophobic leaf surfaces (Fig. 4a), and higher drop rolling efficiency (Fig. 4b). The study indicated that the hydrophobic nature of leaves with moderate trichome density could be more beneficial for sea buckthorn’s adaptability to lower altitudes. The leaf trichomes help maintain the plant water status, while the hydrophobic surface with higher drop rolling efficiency can promote photosynthesis, resulting in better plant growth and development.

Hence, the hypothesis that an assembly of leaf traits enhances water use efficiency and photosynthesis by leveraging anatomical leaf features like trichomes, leaf wettability, and drop rolling efficiency is largely proven. The results further indicated that sea-buckthorn accessions at middle altitudes may capture better physiological adaptability under climate change of depleting water resources and rising temperatures, which was similarly observed by He, et al. ^15^ in *H. rhaimnoides* and *H. sinesis* in China that tend to migrate to and expand in lower altitudes. These accessions having better FS, A, and WUE may promise better economic use and further adaptations to agroecological conditions compared to both higher and lower altitudes.

## Conclusion

Considering commonly used WUE traits of commercially known species like wheat, we assessed, identified, and correlated the diversity of three leaf traits viz. leaf angle, groove type, and leaf rolling, along with leaf contact angle dynamics across five different locations of sea-buckthorn growing regions of Gilgit, Pakistan. Sea buckthorn leaves were found to be hydrophilic with less drop rolling efficiency with exceptions in low-altitude regions where hydrophobic leaves were also found in few accessions. The physiological traits also varied; with transpiration and stomatal conductance higher at highest and lowest altitudes while photosynthesis and water use efficiency higher at the medium altitudes. Overall, we conclude that an assembly of leaf traits with semi-droopy to droopy leaves, light leaf groove, 5–25% inward leaf rolling, moderate leaf trichome density along with hydrophobicity and lower contact angle hysteresis can help in the adaptation of sea-buckthorn accessions under climate change scenario. However, current research is limited due to availability of data only for one site and hence, further research and transplanting experiments to lower altitude regions may be needed to evaluate the effect of climatic differences and thus to simulate potential eco-physiological and morphological effects in sea buckthorn.

## Supplementary Information

Below is the link to the electronic supplementary material.

Supplementary Material 1

## Data Availability

All data generated or analysed during this study are included in this published article [and its supplementary information files]. The raw data files and methods are available from the corresponding author upon reasonable request.
