# Supplementary material for: Altitudinal variation in leaf morphology and functional traits of sea-buckthorn (Hippophae rhamnoides) in Gilgit region, Pakistan
Source: Sci Rep. 2026 Jan 5;16:202. doi: 10.1038/s41598-025-25019-y (PMC12769487; doi:10.1038/s41598-025-25019-y)
Supplement: Supplementary file 1 — Supplementary Material 1 [file 41598_2025_25019_MOESM1_ESM.docx]

**Altitudinal variation in leaf morphology and functional traits of sea-buckthorn (*Hippophae rhamnoides*) in Gilgit region, Pakistan**

Sadia Hakeem^1,2^, Zulfiqar Ali^*,1,3,4^, Muhammad Abu Bakar Saddique^1^, Muhammad Ali Sher^1^, Sabah Merrium^1^, Martin Wiehle^*,5,6^

^1^Institute of Plant Breeding and Biotechnology, MNS University of Agriculture, Multan, Pakistan

^2^Cotton Research Institute, Multan, Ayub Agricultural Research Institute, Faisalabad, Pakistan.

^3^Department of Plant Breeding and Genetics, University of Agriculture, Faisalabad, Pakistan

^4^Programs Department, Islamic Organization for Food Security, Mangilik Yel Ave. 55/21 AIFC, Unit 4, C4.2, Astana, Republic of Kazakhstan

^5^Organic Plant Production and Agroecosystems Research in the Tropics and Subtropics, University of Kassel, Steinstrasse 19, D-37213 Witzenhausen, Germany

^6^Centre for International Rural Development, University of Kassel, Steinstrasse 19, D-37213 Witzenhausen, Germany

^*^ Corresponding email: [zulfiqarpbg@hotmail.com](mailto:zulfiqarpbg@hotmail.com), [wiehle@uni-kassel.de](mailto:wiehle@uni-kassel.de)

**Figure S1: Weather parameters in the month of October 2021 at Hunza, Gilgit, Pakistan.**

Tmax: Maximum temperature (°C), Tmin: Minimum temperature (°C), WS: Wind speed (knots), RH: Relative humidity (%)

**
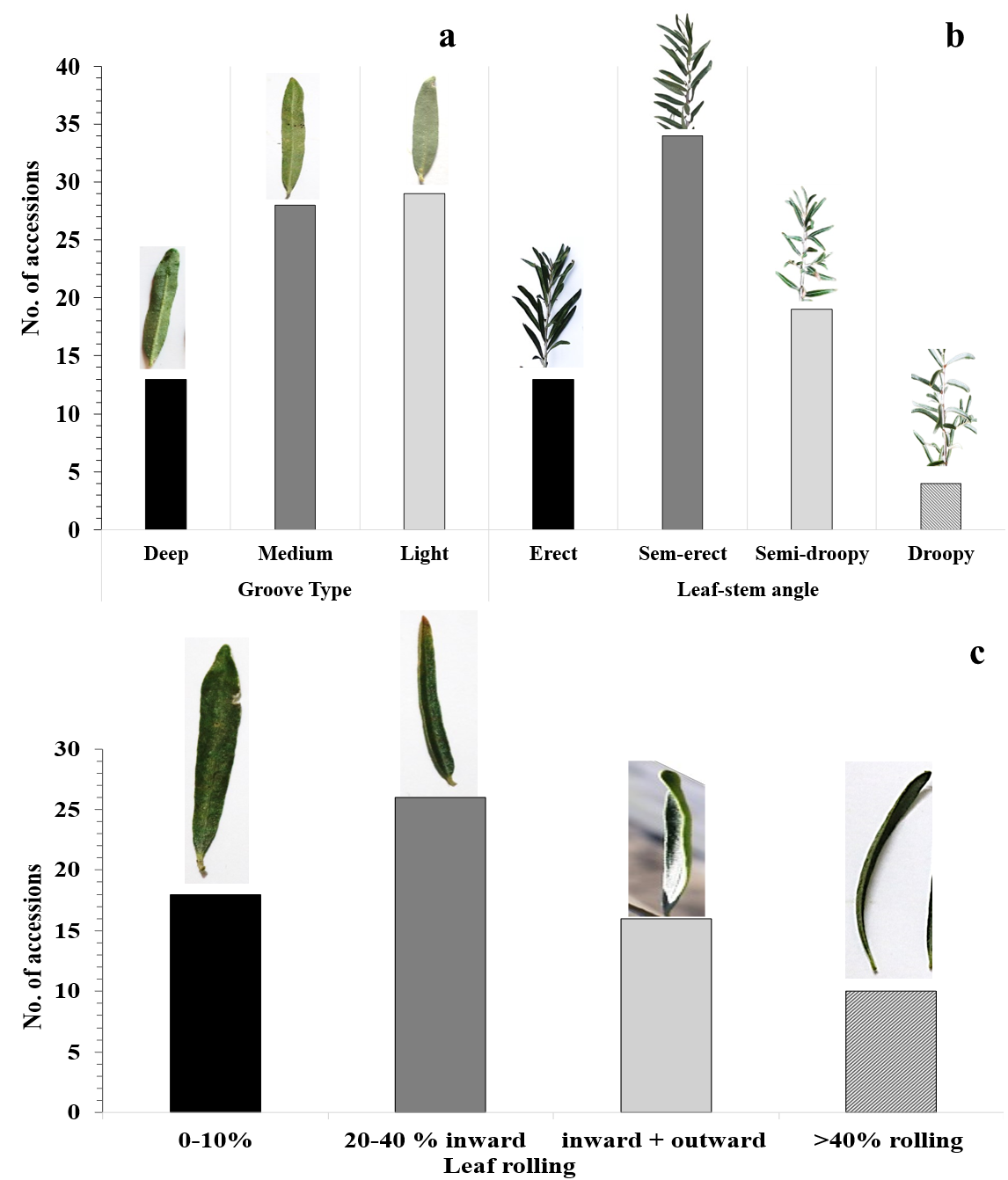
Figure S2: Overall frequency distribution of leaf traits at five locations of Gilgit region.** a. Frequency distribution of groove type, b. Frequency distribution of leaf-stem angle, c. Frequency distribution of leaf rolling.

**
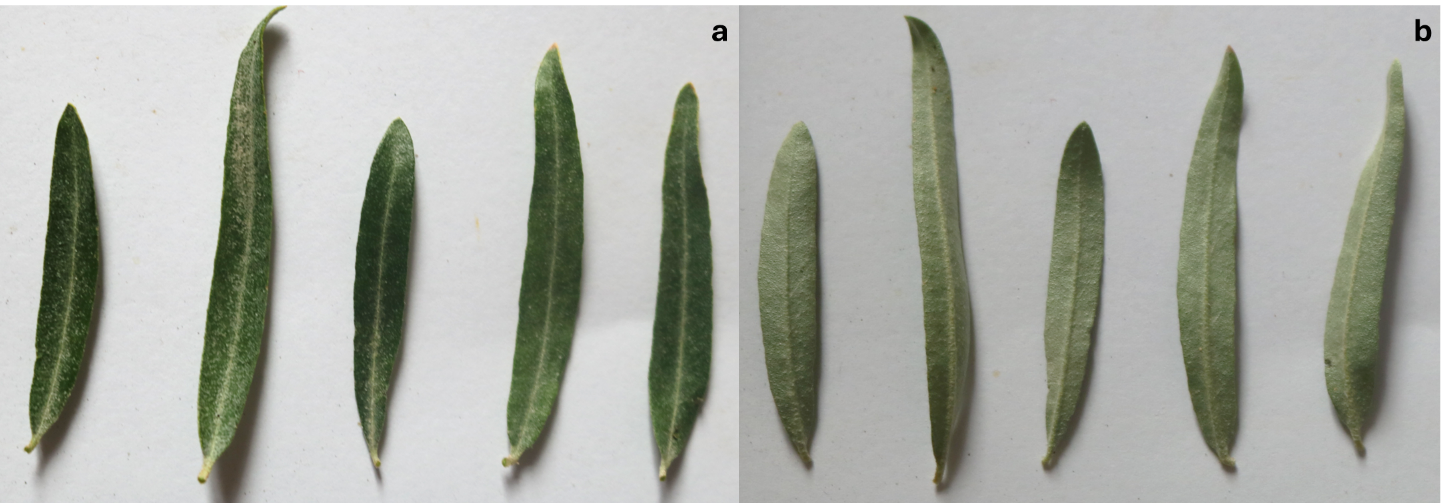
**

**Figure S3: Leaf colour on the adaxial (a) abaxial (b) leaf surface.**

**Table S1:** **Geographical coordinates of the ten sea buckthorn populations studied in Gilgit, Pakistan**

| Village | Location | | |
| --- | --- | --- | --- |
|  | N | E | m.a.s.l |
| Misgar (M) | 36° 47.42 | 074° 46.05 | 2934 |
| Sost (S) | 36° 41.44 | 074° 48.90 | 2887 |
| Passu (P) | 36° 21.18 | 074° 52.41 | 2444 |
| Gulmit (G) | 36° 24.18 | 074° 52.38 | 2523 |
| Shishkat (sh) | 36° 28.84 | 074° 53.05 | 2503 |

**Table S2: Mean square values for the leaf canopy and surface traits for seventy sea-buckthorn accessions**

| SOV | Df | LA | GT | LRA | LR | CAH_ab | CAH_ad |
| --- | --- | --- | --- | --- | --- | --- | --- |
| Replications | 2 | 0.19 | 0.4* | 0.2* | 0.94 | 0.58 | 5.7 |
| Genotypes | 69 | 2.0*** | 1.5*** | 0.7*** | 702.1*** | 102.8*** | 179.9*** |
| Residuals | 138 | 0.12 | 0.11 | 0.04 | 5.27 | 3.11 | 2.43 |

Significance codes: 0.001 ‘***’, 0.01 ‘**’, 0.05 ‘*’, SOV: sources of variation, Df: degree of freedom, LA: leaf angle, GT: groove type, LRA: leaf rolling attitude, LR: leaf rolling, CAH: contact angle hysteresis, ab: abaxial leaf surface, ad: adaxial leaf surface

**Table S2 Contd.: Mean values for the physiological traits and fruit-set for seventy sea-buckthorn accessions**

| SOV | Df | RWC | FS | gs | A | E | WUE |
| --- | --- | --- | --- | --- | --- | --- | --- |
| Replications | 2 | 10.31 | 88.03 | 5.26 | 0.13 | 0.1** | 0.05 |
| Genotypes | 69 | 988.6*** | 610.4*** | 866.0*** | 5.1*** | 0.5*** | 1.5*** |
| Residuals | 138 | 11.10 | 110.74 | 27.74 | 0.23 | 0.01 | 0.03 |

Significance codes: 0.001 ‘***’, 0.01 ‘**’, 0.05 ‘*’, SOV: sources of variation, Df: degree of freedom, RWC: relative water content, FS: fruit-set, gs: stomatal conductance, A: net photosynthesis, E: transpiration, WUE: water use efficiency
